# IFI44L modulates chemoradiotherapy sensitivity and CD8^+^ T cell infiltration in colorectal cancer

**DOI:** 10.3389/fonc.2026.1786425

**Published:** 2026-08-28

**Authors:** Linlin Zheng, Mengjie Li, Ruoqing Yan, Wen Tan

**Affiliations:** State Key Laboratory of Molecular Oncology, Department of Etiology and Carcinogenesis, National Cancer Center/National Clinical Research Center for Cancer/Cancer Hospital, Chinese Academy of Medical Sciences and Peking Union Medical College, Beijing, China

**Keywords:** CCL5, CD8^+^ T cells, chemoradiotherapy sensitivity, colorectal cancer, IFI44L

## Abstract

**Objective:**

Chemoradiotherapy (CRT) represents a cornerstone treatment for colorectal cancer (CRC), yet heterogeneous responses to CRT in treatment response remain a major clinical challenge. Increasing evidence suggests that the tumor immune microenvironment (TME) especially intratumoral CD8^+^ T cells plays an important role in determining CRT efficacy; however, the molecular mechanisms linking tumor-derived factors to immune-mediated treatment sensitivity remain poorly understood. Here, we aimed to identify and characterize regulators of CRT response and the underline mechanisms in CRC.

**Methods:**

Transcriptomic profiling of rectal cancer cohorts was performed to identify genes associated with CRT response, followed by pathway enrichment analyses to explore potential biological mechanisms. The functional role of interferon-induced protein 44-like (IFI44L) was investigated using *in vitro* assays and *in vivo* immunodeficient NSG and immunocompetent BALB/c mouse models to distinguish tumor cell-autonomous from immune-dependent effects. RNA sequencing, immune infiltration analysis, chemotaxis assays, immunofluorescence staining, and enzyme-linked immunosorbent assays (ELISA) were further performed to elucidate the underlying molecular mechanisms.

**Results:**

IFI44L is identified as a candidate gene associated with CRT response through multi-cohort transcriptomic analysis. IFI44L overexpression did not directly alter CRC cell proliferation or CRT response *in vitro*, nor did it affect tumor growth following CRT in immune-deficient NSG mice. In contrast, IFI44L significantly enhanced CRT efficacy in immunocompetent BALB/c models, suggesting that its sensitizing effect was in an immune-dependent manner. Mechanistically, IFI44L promoted the expression and secretion of the T cell-attracting chemokine CCL5, leading to increased intratumoral CD8^+^ T cell infiltration and enhanced CRT response.

**Conclusions:**

Our findings identify IFI44L as an immune-dependent regulator of CRT sensitivity in CRC. By promoting CCL5-mediated CD8^+^ T cell recruitment, IFI44L links tumor-intrinsic molecular alterations with antitumor immune responses and may represent a potential biomarker or therapeutic target for improving CRT efficacy.

## 1 Introduction

Neoadjuvant CRT is recognized as the standard treatment for locally advanced rectal cancer (LARC) (1). Clinical trials comparing conventional neoadjuvant CRT with neoadjuvant radiotherapy alone in patients with LARC have shown that adding chemotherapy to radiotherapy significantly reduces local recurrence rates (2, 3). Results from the RAPIDO, UNICANCER-PRODIGE 23, and STELLAR trials indicated that total neoadjuvant therapy (TNT) yielded superior pathological complete response rates, decreased rates of distant metastasis, and improved survival compared with conventional neoadjuvant CRT (4–6). However, substantial heterogeneity exists in CRT sensitivity. Since non-responders are exposed to unnecessary toxicity and surgical risks without therapeutic benefit, identifying pretreatment biomarkers or essential modulators and elucidating their mechanisms is essential for optimizing clinical decisions, advancing organ preservation strategies, and developing effective approaches to enhance tumor sensitivity to CRT (7, 8).

Current research landscapes on biomarkers predicting CRT sensitivity in CRC includes genomic biomarkers (9), transcriptomic biomarkers (10–12), protein biomarkers (13–25), epigenetic biomarkers (26), circulating tumor DNA based biomarkers (27–31), TME biomarkers (32–37), gut microbiome biomarkers (38–40), patient-derived organoids (41, 42), and artificial intelligence-based predictive models integrating interdisciplinary approaches (43, 44).

The TME has emerged as an important regulator of CRT responses, with accumulating evidence indicating that the presence of intratumoral CD8^+^ T cells plays a pivotal role in determining therapeutic efficacy (32, 45–48). The density of pretreatment intratumoral CD8^+^ tumor-infiltrating lymphocytes (TILs) has been reported to be associated with improved therapeutic outcomes across multiple cancer types, including breast cancer (49), lung cancer (50), head and neck cancer (51), anal cancer (52), and rectal cancer (53–56). A recent study applying single-cell and spatial analyses revealed that TNT reshapes the TME of rectal cancer by expanding interferon-gamma^+^ effector CD8^+^ T cells, highlighting the central role of CD8^+^ T cell-mediated antitumor immunity in TNT outcomes (57). Baseline immune status before treatment, represented by the abundance of CD8^+^ T cell infiltration, has been shown to influence the efficacy of CRT (32, 47). Furthermore, the density of CD4^+^/CD8^+^ T cells in pretreatment rectal cancer biopsies has been reported to predict pathological response to CRT (56). The immune score based on CD3^+^/CD8^+^ T cell density predicts response to neoadjuvant therapy, distant metastasis, and local recurrence, and aids in selecting candidates for “watch-and-wait” strategies (32, 49).

Current studies investigating CD8^+^ T cell related biomarkers for predicting CRT sensitivity in CRC have notable limitations. Differential expression analyses comparing patients with different sensitivities to CRT often fail to yield highly consistent or definitive overlaps in different studies. And the upstream tumor-intrinsic determinants that shape the CD8^+^ T cell associated TME and influence CRT response remain poorly defined. However, despite the overall transcriptomic heterogeneity, IFI44L repeatedly emerged as a gene positively associated with CRT sensitivity across independent transcriptomic analyses. IFI44L is an antiviral component that belongs to the family of interferon-stimulated gene products. These products mediate the type I interferon response, which protects cells from invading viral pathogens (58). IFI44L is involved in apoptosis, signal transduction, epigenetic regulation, and immune responses, serving as a key immune marker in viral and autoimmune diseases (58–64). In oncology, IFI44L has dual roles. It correlates with a favorable prognosis in lung cancer, hepatocellular carcinoma, and osteosarcoma (65–69), by regulating proliferation, migration, apoptosis, stemness, and drug resistance (65–67, 70, 71). IFI44L expression is also correlated with poor prognosis in pancreatic ductal adenocarcinoma and nasopharyngeal carcinoma (72, 73). Notably, the mechanism underlying IFI44L function remains largely unexplored. In lung cancer development, IFI44L directly binds to STAT1 protein, promotes its phosphorylation, activates the JAK/STAT1 signaling pathway, and consequently leads to cancer cell apoptosis (74). In hepatocellular carcinoma, IFI44L affects cancer stemness, metastasis, and drug resistance by regulating the MET/SRC signaling pathway (66). However, the functional role of IFI44L in regulating CRT sensitivity in CRC, especially its potential involvement in shaping TME and influencing CD8^+^ T cell-mediated antitumor responses, remains poorly understood.

GSEA of expression array data from 81 rectal cancer patients revealed significant enrichment of T cell chemotaxis-related pathways in CRT responders, suggesting that immune modulation may contribute to differential CRT sensitivity. However, whether tumor-derived factors regulate CD8^+^ T cell recruitment to influence CRT response in CRC remains unclear. In this study, we aimed to determine whether IFI44L acts as an immune regulator of CRT sensitivity in CRC and to elucidate the underlying mechanisms. We hypothesized that IFI44L enhances CRT efficacy by promoting CD8^+^ T cell infiltration. To address this hypothesis, we integrated transcriptomic analyses, *in vitro* and *in vivo* functional experiments, immune-competent and immune-deficient *in vivo* models, chemotaxis assays, RNA sequencing, and immune profiling approaches. IFI44L was found to enhance CRT efficacy through immune-dependent mechanisms by increasing CCL5 production and promoting CD8^+^ T cell infiltration within tumors. These findings identify IFI44L as a potential biomarker and regulator of CRT sensitivity in CRC.

## 2 Materials and methods

### 2.1 Patient cohorts

The cohort consisted of 81 patients with locally advanced rectal cancer enrolled between January 2006 and June 2013 at the Cancer Hospital, Chinese Academy of Medical Sciences (Beijing, China), as previously described (18). All patients received preoperative concurrent chemoradiotherapy, and pretreatment biopsy specimens were collected for subsequent analysis. The eligibility criteria, treatment regimens, and response evaluation criteria have been described in detail in our previous publication (18). Clinical characteristics of these patients are summarized in **Table 1**. This study received approval from the Institutional Review Board of the Cancer Institute, Chinese Academy of Medical Sciences (IRB no. 23/088–3827). Response to CRT was evaluated per the tumor regression classification illustrated by Mandard et al. (75). Four additional independent public datasets were included: one CRC dataset from the ‘The Cancer Genome Atlas’ (TCGA-CRC, n = 618), downloaded from Genomic Data Commons (GDC) Data Portal portal.gdc.cancer.gov, two expression array datasets from Gene Expression Omnibus (GEO) (GSE150082, n = 39 and GSE209746, n = 114), one single-cell RNA sequencing (scRNA-seq) dataset from GEO (GSE205506, n = 22), downloaded from the GEO Accession viewer https://www.ncbi.nlm.nih.gov/geo/query/acc.cgi.

**Table 1:** Clinical characteristics of 81 patients with rectal cancer and their association with response to neoadjuvant chemoradiotherapy.

Clinical characteristics	All samples (%)	Responder (TRG1 + 2)	Moderate-responder (TRG3)	Non-responder (TRG4 + 5)	*P* value
Number	81 (100.0)	30 (37.0)	37 (45.7)	14 (17.3)	
Age (Year)					0.732
<54	40 (49.4)	16 (53.3)	17 (45.9)	7 (50.0)	
≥54	41 (50.6)	14 (46.7)	20 (54.1)	7 (50.0)	
Gender					0.912
Male	54 (66.7)	20 (66.7)	25 (67.6)	9 (64.3)	
Female	27 (33.3)	10 (33.3)	12 (32.4)	5 (35.7)	
Stage					0.410
II	19 (23.5)	8 (26.7)	9 (24.3)	2 (14.3)	
III	62 (76.5)	22 (73.3)	28 (75.7)	12 (85.7)	
KPS					0.177
80-89	34 (42.0)	16 (53.3)	13 (35.1)	5 (35.7)	
90-100	47 (58.0)	14 (46.7)	24 (64.9)	9 (64.3)	
DFS					0.003
Alive	42 (51.9)	23 (76.7)	14 (37.8)	5 (35.7)	
Dead	39 (48.1)	7 (23.3)	23 (62.2)	9 (64.3)	

*P* value is calculated by chi-square test or Fisher’s exact test.

KPS, Karnofsky performance score.

DFS, Disease-free survival.

### 2.2 Bioinformatics analysis

Limma package (version 3.56.2) was used to exert differential expression analysis. Differentially expressed genes (DEGs) were identified using a *P* value < 0.05 and |log2Fold Change (FC)| > 1, with non-responders as the control group. Significantly enriched pathways were identified by GSEA, GO and KEGG analysis using the Custer Profiler package (version 4.8.3). CIBERSORT was performed using the CIBERSORT R script (version 1.03), whose core principle was a regression method based on nu-support vector regression (v-SVR) and a predefined immune cell signature gene matrix (LM22) was used as a reference for the algorithm. The Cell-Cell Communication Analysis Toolkit was used for CellChat analysis using the CellChat package (version 1.6.1).

### 2.3 Cell lines and reagents

RPMI-1640 medium was used to culture CT26 and Jurkat cells, while RKO and HCT116 cells were maintained in DMEM. Both media were supplemented with 10% fetal bovine serum (FBS). All cells were incubated at 37 °C in a humidified atmosphere containing 5% CO_2_. 5-Fluorouracil (5-FU; HY-90006) and Oxaliplatin (HY-17371) obtaining from MedChemExpress (Monmouth Junction, NJ, USA) were dissolved using dimethyl sulfoxide.

### 2.4 Generation of CRC cell lines stably overexpressing IFI44L

Lentivirus used to generate stable IFI44L-OE was purchased from GeneChem (Shanghai, China). The GV492 vector plasmid used followed the order: Ubc-MCS-3FLAG-CBh-gcGFP-IRES-puromycin.

### 2.5 Quantitative real-time polymerase chain reaction analysis

Relative gene expression was determined through reverse transcription quantitative polymerase chain reaction (RT-qPCR) using SYBR Green (RR820A; TaKaRa, Dalian, China). Total RNA was extracted using the RNA Quick Purification Kit (RN001; ES Science, Shanghai, China). Reverse transcription was subsequently performed for cDNA synthesis using the PrimeScript RT Master Mix (RR0036A; TaKaRa, Dalian, China). The primer sequences used in this study are listed in **Supplementary Table 1**.

### 2.6 Western blot analysis

Cells were lysed in RIPA buffer containing PMSF and phosphatase inhibitors, followed by centrifugation (12,000 × g, 15 min, 4 °C). Protein concentrations were determined using a BCA assay. Equal amounts of protein were resolved by SDS–PAGE, transferred to PVDF membranes, and subjected to immunoblotting with anti-IFI44L (Cell Signaling Technology) and anti-VINCULIN (Proteintech) antibodies. Signals were detected using enhanced chemiluminescence and imaged with an Amersham Imager 600.

### 2.7 Xenograft tumor model

Animal procedures were carried out following approval from the Institutional Animal Care and Use Committee of the Chinese Academy of Medical Sciences (Approval No. NCC2022A061). Five-week-old male NSG mice (Beijing HFKBIOSCIENCE Co.) received subcutaneous injections of either IFI44L-OE or Vector RKO and HCT116 cells (2×10^6^ cells). In a separate experiment, age-matched male BALB/c mice (Beijing HFKBIOSCIENCE Co.) were injected subcutaneously with Ifi44l-OE or Vector CT26 cells (2×10^6^ cells). Mice were then randomly assigned to either a control (vehicle) group or a CRT treatment group. The CRT group underwent a 2-week regimen of radiotherapy (4 Gy) combined with intraperitoneal chemotherapy [5-FU (20 mg/kg) with Oxaliplatin (5 mg/kg)], whereas control mice received isovolumetric injections of normal saline on an equivalent schedule (**Supplementary Figure 1**). A monoclonal anti–mouse Cd8α antibody (Bio X Cell) was injected intraperitoneally (200 μg per animal). The first injection was given 2 days before the initiation of treatment, followed by repeated injections every 5 days to maintain Cd8^+^ T cell depletion. Control mice received an equal dose of isotype-matched IgG2b antibody.

### 2.8 Multiplex immunofluorescence staining analysis

Multiplex immunofluorescence staining was carried out on using a multiplex immunohistochemistry system based on Opal fluorophores (PANOVUE). After deparaffinization, rehydration, and antigen retrieval, sections were incubated with a panel of primary antibodies targeting PanCK (Proteintech), IFI44L (BOSTER), and CD8A (Cell Signaling Technology). Signal amplification was accomplished through sequential rounds of HRP-conjugated secondary antibody incubation and TSA-based fluorophore deposition (Opal 650, 570, 480, 620). Following DAPI counterstaining, spectral images were acquired with a NanoZoomer scanner. Quantitative analysis of staining intensity, averaged across three random fields per sample, was performed in a blinded manner using ImageJ software.

### 2.9 *In vitro* T cell chemotaxis assay

Conditioned media (CM) were harvested from serum-starved IFI44L-OE, Vector RKO, or HCT116 cells cultured in six-well plates. For the migration assay, CM was placed in the lower chambers of 24-well transwell plates (5 μm pore size). Jurkat cells (3×10^5^) were seeded into the upper chambers (n = 3 per group). After 24–48 h of co-culture, cells in the lower chamber were resuspended in PBS, and quantified. Separately, CD8^+^ T cells isolating from BALB/c mouse spleens were cultured in complete RPMI-1640 medium supplemented with 10% FBS, 2 mM L-glutamine, 10 mM HEPES, 1 mM sodium pyruvate, 50 μM 2-mercaptoethanol, and 30–100 U/mL murine IL-2, and were activated using anti-CD3/CD28-conjugated beads. For chemotaxis assessment, activated CD8^+^ T cells (1×10^5^) were placed in the upper chamber, while the lower chamber contained CT26-derived CM. Migrated cells were collected after 12 h, resuspended in PBS, and counted.

### 2.10 RNA sequencing and bioinformatics analysis

Total RNA was extracted from cells using TRIzol reagent (Invitrogen). Library sequencing was conducted on an Illumina NovaSeq 6000 platform. For the identification of differentially expressed genes, we employed the DESeq2 package (version 1.40.2).

### 2.11 Enzyme-linked immunosorbent assay

The concentrations of CCL5 in CM were quantified using ELISA assay kits (Proteintech) in accordance with the manufacturer’s instructions.

### 2.12 Cell viability and colony formation assays

RKO or HCT116 cells were plated in 96-well or 6-well plates at specified densities in complete medium, with or without concurrent CRT (5 μg/mL 5-FU, 1 μg/mL Oxaliplatin, 2 Gy). Cell viability was measured daily using CCK-8 (Dojindo Lab) according to the manufacturer’s protocol (1.5 h incubation at 37 °C; OD_450_ detection). For IC_50_ determination, cells were exposed to drug serial dilutions for 48 h before CCK-8 measurement. For clonogenic assay, after 7 days of culture, formed colonies were fixed and then stained with crystal violet (0.5%), and quantified using ImageJ software.

### 2.13 Statistical analysis

Statistical analyses were performed using R (v4.3.1) and GraphPad Prism (v9.5). Two-tailed Student’s *t*-test was used for normally distributed data. The Wilcoxon rank-sum test was employed for non-normally distributed data to determine the significance of differences between two groups. Spearman’s rank correlation coefficient was applied to assess associations between variables.

## 3 Results

### 3.1 IFI44L is identified as a candidate gene associated with CRT response through multi-cohort transcriptomic analysis

In our previous three studies, we aimed to identify modulators related to sensitivity of CRC to CRT (18, 76, 77). A gene expression profiling assay was performed in 81 patients with CRC who received preoperative CRT followed by surgery and exhibited different treatment responses.

**Figure 1:**
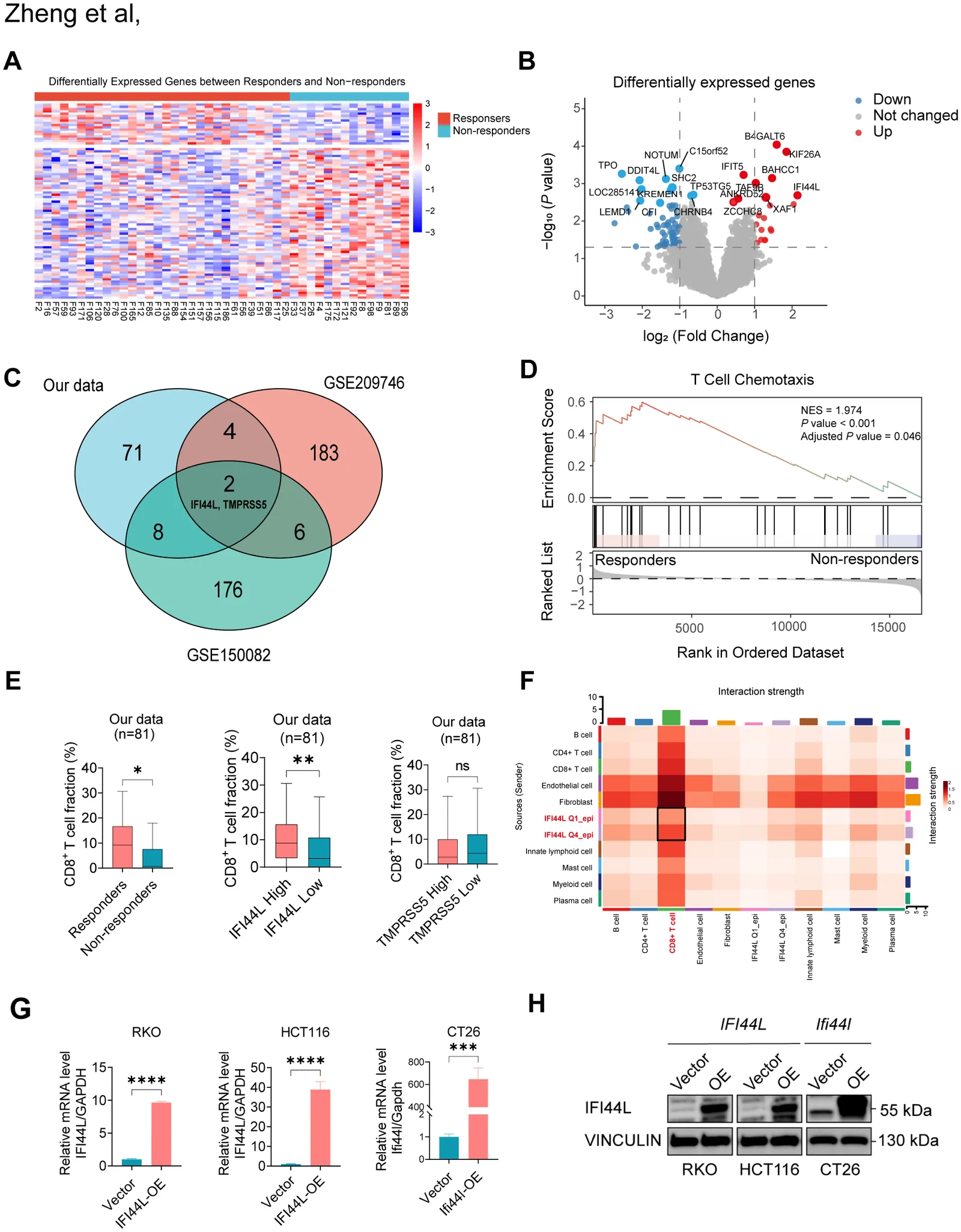
IFI44L is a key gene related to chemoradiotherapy sensitivity in colorectal cancer. **(A)** Heatmap plot of DEGs between the responders and non-responders. **(B)** Volcano plot of DEGs between the responders and non-responders. The top 20 genes ranked by *P* value are marked with gene symbol. **(C)** The intersection of DEGs from the 81-sample cohort (our data), GSE150082 and GSE209746. **(D)** GSEA was performed on the 81-sample cohort (our data) between responders and non-responders. **(E)** Proportion of infiltrating CD8^+^ T cells in responders or non-responders, IFI44L high or low group and TMPRSS5 high or low group were calculated via CIBERSORT immune infiltration analysis in responders and non-responders. **(F)** Interaction heatmap summarizing CellChat-inferred ligand–receptor communication across cell types. **(G)** Relative mRNA levels of IFI44L among Vector and IFI44L-OE RKO, HCT116, and CT26 cell lines were tested by RT-qPCR. **(H)** Protein levels of IFI44L among Vector and IFI44L-OE RKO, HCT116, and CT26 cell lines were tested by Western blot. *P < 0.05; **P < 0.01; ***P < 0.001; ****P < 0.0001, and ns, not significant.

Differential expression analysis was performed between 30 responders and 14 non-responders, identifying 84 DEGs (**Figures 1A, B**; **Supplementary Table 2**). To further identify key genes related to CRT efficacy, we analyzed two additional CRC transcriptomic datasets, GSE150082 (n = 39) and GSE209746 (n = 114), with results shown in **Supplementary Tables 3**, **S4**. Different FC thresholds were applied to assess the consistency of gene screening and the reliability of candidate gene identification (**Supplementary Table 5**). Intersecting of DEGs across all datasets identified IFI44L and TMPRSS5 as candidate genes (**Figure 1C**). Compared with non-responders, responders showed significant enrichment of the T cell chemotaxis pathway (NES = 1.974, *P* < 0.001, adjusted *P* = 0.045; **Figure 1D**) and a higher proportion of CD8^+^ T cells (*P* = 0.031, **Figure 1E**).

To link the two candidate genes with CD8^+^ T features and CRT response, 81 patients were stratified into high or low-expression groups based on the median expression of the candidate genes. CIBERSORT immune infiltration analysis revealed that the IFI44L high-expression group had significantly higher CD8^+^ T cell infiltration than the low-expression group (*P* = 0.006), whereas no significant difference was observed between TMPRSS5 expression groups (*P* = 0.376) (**Figure 1E**). Furthermore, cancer cells in the single-cell dataset GSE205506 were stratified into IFI44L High (IFI44L_Q4_epi) and IFI44 Low (IFI44L_Q1_epi) groups based on expression quartiles, CellChat analysis was subsequently performed separately on the two groups. The IFI44L-high epithelial cells exhibited stronger interactions with CD8^+^ T cells than the IFI44L-low group (**Figure 1F**).

### 3.2 IFI44L overexpression enhances chemoradiotherapy sensitivity in colorectal cancer in a CD8^+^ T cell-dependent manner

**Figure 2:**
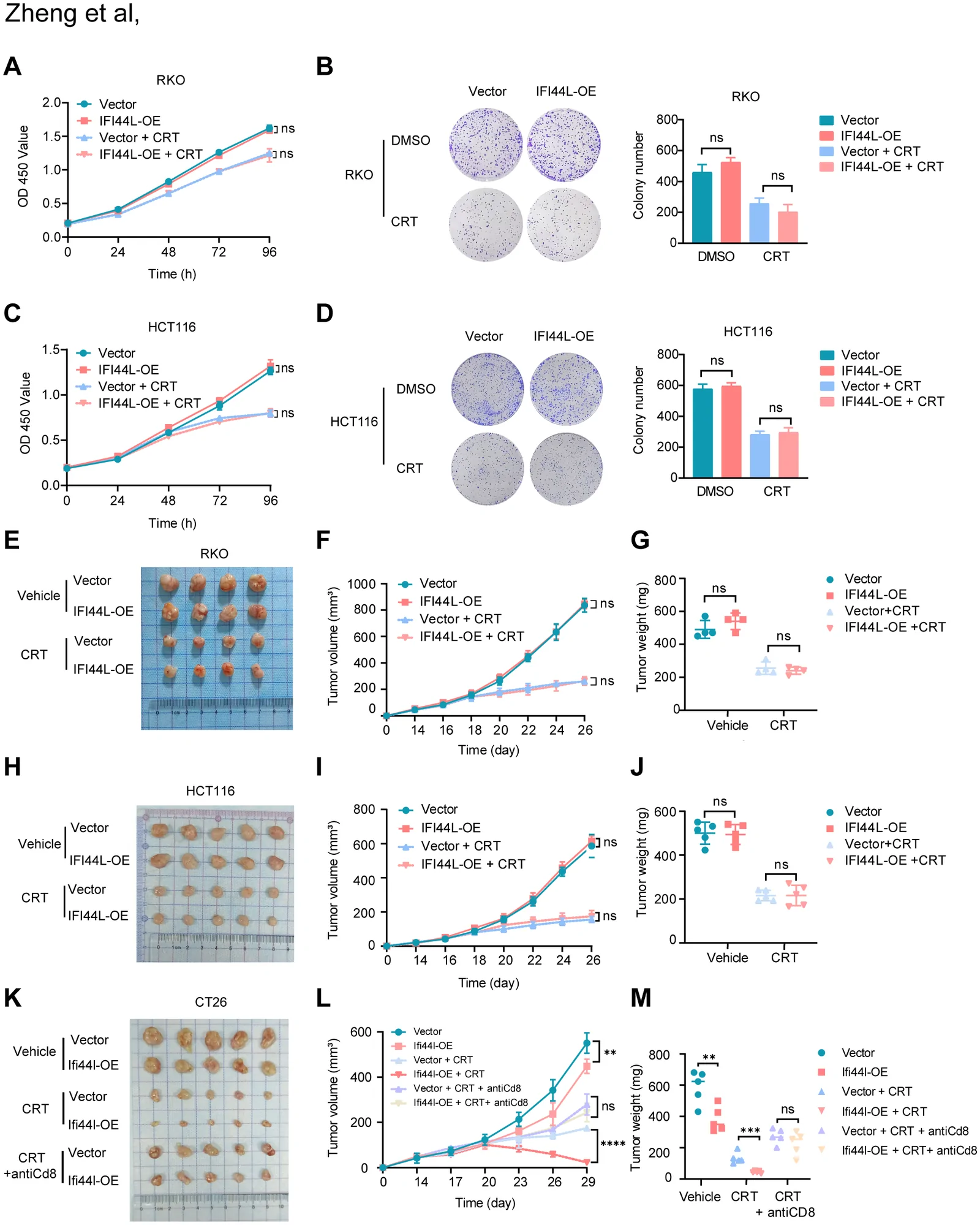
Overexpression of IFI44L enhances the sensitivity of colorectal cancer to chemoradiotherapy in a CD8^+^ T cell–dependent manner. **(A, B)** CCK-8 proliferation assay **(A)** and colony-formation assay **(B)** results of IFI44L-OE RKO and Vector RKO cells under untreated or CRT treated conditions. **(C, D)** CCK-8 proliferation assay **(C)** and colony-formation assay **(D)** results of IFI44L-OE HCT116 and Vector HCT116 cells under untreated or CRT treated conditions. **(E–G)** Representative images of subcutaneous tumors **(E)**, proliferation curves **(F)**, and tumor weights **(G)** in severe combined immunodeficient mice bearing IFI44L-OE RKO or Vector RKO tumors. **(H–J)** Representative images of subcutaneous tumors **(H)**, proliferation curves **(I)**, and tumor weights **(J)** in severe combined immunodeficient mice bearing IFI44L-OE HCT116 or Vector HCT116 tumors. **(K–M)** Representative images of subcutaneous tumors **(K)**, proliferation curves **(L)**, and tumor weights **(M)** in immunocompetent BALB/c mice bearing Ifi44l-OE CT26 or Vector CT26 tumors. **P < 0.01; ***P < 0.001; ****P < 0.0001; and ns, not significant.

Based on the preceding analyses, IFI44L was selected as a key candidate for further investigation regarding its role in CRT response. Stable IFI44L-OE CRC cell lines (IFI44L-OE RKO, IFI44L-OE HCT116 and Ifi44l-OE CT26) were generated and validated using RT-qPCR and WB (**Figures 1G, H**). As previous studies have reported that IFI44L can enhance chemosensitivity in lung cancer cells by apoptosis (74), we examined whether IFI44L-OE affects the response of CRC cell lines to CRT *in vitro*. Compared with the Vector group, IFI44L-OE group did not gain a different proliferative capacity nor enhanced sensitivity to radiotherapy or chemotherapy in RKO and HCT116 CRC cells (**Supplementary Figures 2A–F**). When radiotherapy and chemotherapy were combined to mimic concurrent CRT, IFI44L-OE similarly did not affect cell proliferation or CRT sensitivity in CRC cell line (**Figures 2A–D**). These findings indicate that IFI44L-OE does not influence CRC cell proliferation or their response to CRT *in vitro*.

Next, to determine whether this sensitizing effect depends on the immune system, we inoculated severe combined immunodeficient NSG mice with IFI44L-OE RKO or Vector RKO cells (**Figure 2E**). At the experimental endpoint, tumor volume also did not differ significantly between IFI44L-OE RKO and Vector RKO tumors in either untreated (*P* = 0.761) or treatment groups (*P* = 0.899) (**Figure 2F**). In the untreated groups, tumor weight did not significantly differ between IFI44L-OE RKO and Vector RKO tumors (*P* = 0.225). Similarly, no significant difference was observed in the treatment groups (*P* = 0.513) (**Figure 2G**). Subcutaneous xenografts in NSG mice were established using IFI44L-OE and Vector HCT116 cell lines (**Figure 2H**). At the observation endpoint, in the untreated groups, tumor volumes did not significantly differ between IFI44L-OE (614.42 ± 32.12) and Vector (586.40 ± 66.72) HCT116 tumors (*P* = 0.422). Tumor volumes in the IFI44L-OE (174.12 ± 34.44) and Vector (155.60 ± 17.18) HCT116 groups showed no significant difference (*P* = 0.313) (**Figure 2I**). No significant difference was observed in tumor weight between the IFI44L-OE (494.20 ± 45.32) and Vector (500.33 ± 50.22) HCT116 groups in the untreated condition (*P* = 0.844). Similarly, in the treated conditions, tumor weights in the IFI44L-OE (216.76 ± 46.67) and Vector (216.12 ± 23.94) HCT116 groups showed no significant difference (*P* = 0.979) (**Figure 2J**). Overall, these results indicated that IFI44L-OE does not alter CRT sensitivity in immunodeficient mice.

When Ifi44l-OE CT26 and Vector CT26 cell lines were implanted into immunocompetent BALB/c mice (**Figure 2K**), at the observation endpoint, tumor volume in the untreated Ifi44l-OE CT26 group (447.94 ± 31.62) was significantly smaller than that in the Vector CT26 group (550.06 ± 45.43) (*P* = 0.003). Likewise, in the treated groups, tumor volume in the Ifi44l-OE CT26 group (23.00 ± 3.19) showed a pronounced reduction compared with the Vector CT26 group (174.86 ± 11.56) (*P* < 0.001) (**Figure 2L**). Tumor weight in the untreated Ifi44l-OE CT26 group (381.46 ± 86.63) was significantly lower than that in the Vector CT26 group (588.72 ± 100.69) (*P* = 0.008). In the treated groups, tumor weight in the Ifi44l-OE CT26 group (44.60 ± 7.10) was also significantly lower than that in the Vector CT26 group (133.38 ± 32.33) (*P* < 0.001) (**Figure 2M**). Notably, depletion of Cd8^+^ T cells in BALB/c mice using an anti-mouse Cd8a monoclonal antibody abolished the treatment-sensitizing effect of IFI44L-OE. Under these conditions, at the observation endpoint, tumor volume in the Ifi44l-OE CT26 group (243.88 ± 40.63) did not significantly differ from that in the Vector CT26 group (278.52 ± 46.35) (*P* = 0.244) (**Figure 2L**). Similarly, no significant difference occurred in tumor weight between the Ifi44l-OE CT26 group (227.58 ± 71.47) and the Vector CT26 group (272.94 ± 45.08) (*P* = 0.282) (**Figure 2M**). Collectively, these findings demonstrated that IFI44L-OE enhances the sensitivity of immunocompetent BALB/c mice to CRT, and that this sensitizing effect is dependent on the presence of Cd8^+^ T cells.

### 3.3 IFI44L overexpression promotes the production of CD8^+^ T cell-attracting chemokine CCL5

**Figure 3:**
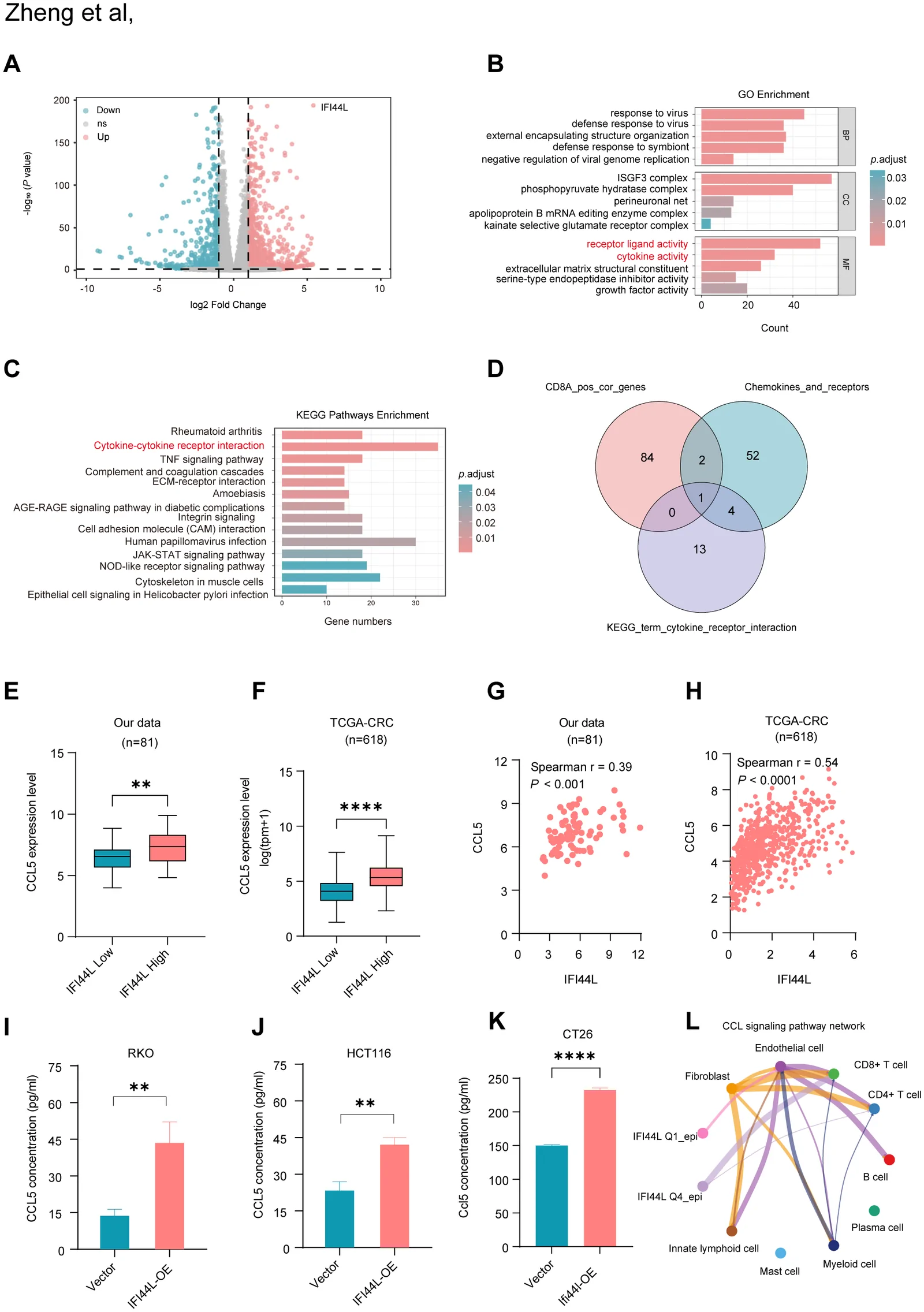
Overexpression of IFI44L promotes the production of CD8^+^ T cell attracting chemokine CCL5. **(A)** Volcano plot of differentially expressed genes identified by RNA-seq analysis between IFI44L-OE and Vector RKO cell lines. **(B)** GO enrichment analysis of upregulated genes in IFI44L-OE RKO cells. **(C)** KEGG pathway enrichment analysis of upregulated genes in IFI44L-OE RKO cells. **(D)** Intersection analysis of molecules positively correlated with CD8A in the 81-sample microarray dataset, genes enriched in the KEGG cytokine pathway, and chemokines and chemokine receptor family gene sets, identifying the chemokine CCL5. **(E, F)** Comparison of CCL5 mRNA expression levels between patients with high and low IFI44L expression in the 81-sample cohort (our data) **(E)** and the TCGA-CRC cohort **(F)**. **(G, H)** Spearman correlation analysis between IFI44L and CCL5 expression levels in the 81-sample cohort (our data) **(G)** and the TCGA-CRC cohort **(H)**. **(I, J)** ELISA measurement of CCL5 concentrations in conditioned media from control and IFI44L-OE RKO cells **(I)** and HCT116 cells **(J)**. **(K)** Ccl5 concentration in conditioned media from control and Ifi44l-OE CT26 cells. **(L)** IFI44L quartiles were used to stratify cancer cells in the GSE205506 dataset into IFI44L High and IFI44L Low groups. The CCL chemokine ligand–receptor interactions among cell types cells were calculated with CellChat analyses. **P < 0.01; ****P < 0.0001.

To elucidate underlying mechanisms by which IFI44L enhanced sensitivity to CRT, RNA sequencing was performed on IFI44L-OE and vector RKO (n = 5 per group) (**Figure 3A**; **Supplementary Table 6**). Differential expression analysis of the resulting expression matrices was followed by GO and KEGG enrichment analyses, revealing that the cytokine-cytokine receptor interaction pathway was significantly enriched in IFI44L-OE RKO cells (**Figures 3B, C**). Chemokines are a family of cytokines and have been widely reported to be associated with immune cell chemotaxis and infiltration. Therefore, we intersected molecules positively correlated with CD8A in our array dataset, genes enriched in the KEGG cytokine pathway, and gene sets of chemokines and their receptors, which led to the identification of the chemokine CCL5 (**Figure 3D**). Next, the 81 patients with rectal cancer were stratified into high- or low-IFI44L expression groups. A significant increase in CCL5 mRNA expression was observed in the IFI44L High group versus the IFI44L Low group (*P* = 0.007) (**Figure 3E**). This finding was further validated in the TCGA-CRC cohort (*P* < 0.001) (**Figure 3F**). Correlation analyses demonstrated a significant positive correlation between IFI44L and CCL5 expression in both our array dataset (*P* < 0.001, r = 0.40, 95% CI = 0.189–0.57) and the TCGA-CRC cohort (*P* < 0.0001, r = 0.54, 95% CI = 0.48–0.60) (**Figures 3G, H**). To experimentally validate these findings, CM were collected from IFI44L-OE and negative lentiviral control RKO and HCT116 cell lines, and CCL5 concentrations were measured using ELISA. Compared with CM from Vector control cells, CCL5 concentrations were significantly increased in CM from IFI44L-OE RKO cells (*P* = 0.005) (**Figure 3I**). This result was further confirmed in HCT116 cells (*P* = 0.002) (**Figure 3J**), as well as in the murine CT26 cell line (*P* < 0.001) (**Figure 3K**). CellChat analysis revealed that IFI44L High cancer cells exhibited stronger C-C motif chemokine ligand (CCL) signaling toward CD8^+^ T cells compared with the IFI44L Low group (**Figure 3L**), suggesting that cancer cells with high IFI44L expression may have the potential to promote CD8^+^ T cell chemotaxis and infiltration by enhancing chemokine signaling.

### 3.4 IFI44L overexpression enhances chemoradiotherapy sensitivity in immunocompetent BALB/c mice by promoting CD8^+^ T cell recruitment and infiltration

**Figure 4:**
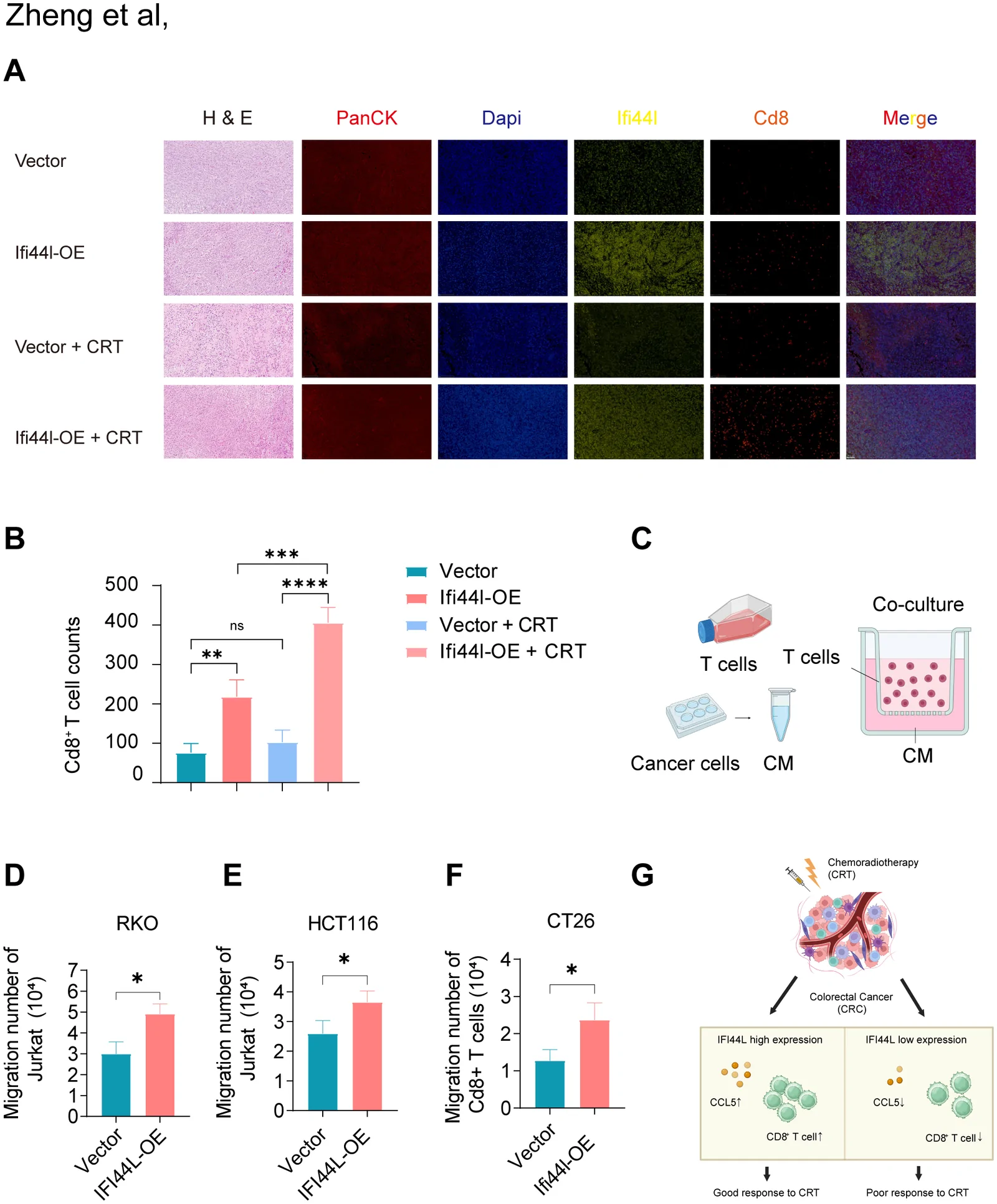
Overexpression of IFI44L promotes the chemotactic recruitment and infiltration of CD8^+^ T cells. **(A, B)** Multiplex immunofluorescence images **(A)** and quantification **(B)** of Cd8^+^ T cell infiltration in subcutaneous tumors from BALB/c mice bearing Ifi44l-OE CT26 or Vector CT26 tumors. Three representative fields were selected, and the average value was used as the measurement for each tumor sample. **(C)** Schematic diagram of the co-culture model using CRC cells and Jurkat T cells and CD8^+^ T cells. **(D, E)** Quantification of migrated Jurkat T cells in the lower chamber after co-culture with RKO cells **(D)** or HCT116 cells **(E)**. **(F)** Quantification of migrated Cd8^+^ T cells in the lower chamber after co-culture with Ifi44l-OE CT26 or Vector CT26 cells. **(G)** Working model. CRC patients with high IFI44L expression exhibit increased sensitivity to CRT. IFI44L enhances the sensitivity of CRC to CRT via modulating the production of the CD8^+^ T cell-attracting chemokine CCL5, thereby facilitating CD8^+^ T cell recruitment and infiltration. *P < 0.05; **P < 0.01; ***P < 0.001; ****P < 0.0001, and ns, not significant.

Previous studies indicated that pretreatment CD8^+^ T cell infiltration correlates with favorable CRT outcomes (32, 47), consistent with our GSEA and immune infiltration analyses. To further elucidate the mechanism by which IFI44L enhances CRT treatment sensitivity, we performed multiplex immunofluorescence staining of BALB/c tumor sections (**Figure 4A**). IFI44L-OE significantly increased Cd8^+^ T cell abundance in the treatment group than that in the untreated group (*P* < 0.001). Conversely, in Vector CT26-bearing mice, Cd8^+^ T cell counts did not differ significantly before and after treatment (*P* = 0.210). Moreover, in both the untreated (*P* = 0.001) and treated groups (*P* < 0.001), Cd8^+^ T cell infiltration was markedly higher in Ifi44l-OE CT26 tumors than in vector CT26 tumors (**Figure 4B**). These findings suggested that IFI44L-OE increased Cd8^+^ T cell infiltration, particularly baseline (pretreatment) infiltration, aligning with our previous bioinformatics analysis.

We then established tumor-T-cell co-culture models (**Figure 4C**). CRC cell lines were co-cultured with Jurkat T cells, and the migrated Jurkat cells were collected and counted. Compared with Vector RKO cells, IFI44L-OE RKO cells significantly increased chemotaxis of Jurkat T cells (*P* = 0.011) (**Figure 4D**). Similar results were observed in HCT116 cell line (*P* = 0.035) (**Figure 4E**). When Ifi44l-OE CT26 or Vector CT26 cells were co-cultured with Cd8^+^ T cells isolated from BALB/c mice, the number of Cd8^+^ T cells migrating to the lower chamber was also significantly higher in the Ifi44l-OE CT26 co-culture system than in the Vector group (*P* = 0.026) (**Figure 4F**). These results indicated that IFI44L promotes CD8^+^ T cell chemotaxis by enhancing T cell chemotaxis.

## 4 Discussion

This study identified IFI44L as a key molecule associated with CRT response in CRC through integrated transcriptomic analyses of responders and non-responders. IFI44L significantly enhanced treatment efficacy and suppressed tumor growth in immunocompetent mice. IFI44L-OE modulates the production of CD8^+^ T cell attracting chemokine CCL5 and enhances CD8^+^ T cell infiltration (**Figure 4G**).

IFI44L reportedly exerts tumor-promoting or tumor-suppressive effects across multiple cancers; however, its association with CRC and CRT sensitivity has not been previously investigated. Our results indicated that IFI44L-OE did not directly affect tumor cell proliferation. This differs from findings in osteosarcoma (67), highlighting that cancer-type heterogeneity may influence the biological roles of IFI44L. Interestingly, although IFI44L-OE did not directly affect tumor cell intrinsic sensitivity to CRT *in vitro* or in immunodeficient models, it enhanced CRT sensitivity in immunocompetent BALB/c mice. This extends current knowledge by linking IFI44L to immune-mediated enhancement of CRT sensitivity. The TME is increasingly appreciated as a critical factor influencing responses to CRT (32, 45–47), and CD8^+^ T cell infiltration has been shown to be a critical determinant (32, 48). Through IFI44L-OE and rescue experiments, we found that IFI44L-OE increased the sensitivity of CRC to CRT in a CD8^+^ T cell dependent manner.

Multiple studies have highlighted the crucial role of chemokines in not only diagnosis but also shaping antitumor immunity and enhancing therapy response (78–82). Combining strategies that promote the chemotaxis and infiltration of effector immune cells with conventional cancer therapies may represent an effective approach to enhance antitumor efficacy. For instance, IL-33–induced C-X-C motif chemokine ligand 10 increases 5-FU chemosensitivity in CRC (83). Our results extended current knowledge by linking IFI44L to CCL5, which is the main chemokine involved in CD8^+^ T cell chemotaxis. However, the precise mechanism by which IFI44L promotes the secretion of CCL5 remains to be further investigated.

Receiver operating characteristic curve analysis was performed and IFI44L expression demonstrated a favorable ability to discriminate responders from non-responders, with an area under the curve of 0.774 (95% CI: 0.633–0.914) (**Supplementary Figure 3**), suggesting that IFI44L expression may have potential value as a predictive biomarker for CRT sensitivity. Since IFI44L was initially identified based on microarray expression profiling data, we propose that its expression could be further evaluated in clinical settings using qPCR-based detection in future studies. Furthermore, the potential value of integrating IFI44L expression with established biomarkers, including Immuno-score and other clinically recognized biomarkers such as tumor mutational burden, remains to be investigated.

This study has some limitations. First, as a study investigating a single molecular biomarker or a potential therapeutic strategy, our study has inherent limitations, especially since the current NCCN guidelines rely on established biomarkers such as MSI status, KRAS mutations, and BRAF mutations to guide treatment decisions. More comprehensive clinical cohort information including MSI/MSS status and KRAS/NRAS/BRAF mutations will be needed in future studies, not only to adequately account for potential confounding factors that may contribute to variability in treatment response and thereby improve the reliability and robustness of the analyses, but also to further investigate the potential associations between IFI44L and other molecular pathways or clinically recognized biomarkers recommended in the NCCN guidelines. Second, since IFI44L was found to enhance CRT sensitivity and its basal expression level was relatively low in CRC cell lines, we performed gain-of-function experiments by overexpressing IFI44L, whereas loss-of-function assays were not conducted. Third, The HCT116 and RKO cell lines used in this study are both microsatellite instability-high (MSI-H) cell lines, which may introduce certain cell line biases. The heterogeneity across CRC subtypes, particularly between microsatellite-stable and microsatellite instability-high tumors, was not fully addressed in this study. Whether IFI44L exerts similar immunomodulatory and sensitizing effects across different molecular subtypes remains unelucidated, given their distinct immune landscapes and treatment responses. Fourth, this study primarily focused on the effect of IFI44L on CD8^+^ T cells and its role in modulating CRT sensitivity based on the results of GSEA and CIBERSORT immune infiltration analysis. However, the potential contributions of other cellular components, such as fibroblasts and endothelial cells, warrant further investigation given the complexity of the TME and our previous findings on its role in gastrointestinal cancer progression and treatment sensitivity (84). Fifth, future studies using humanized mouse models or patient-derived xenografts models will be valuable to further validate the clinical relevance of IFI44L-mediated CRT sensitization and its interaction with the TME. Finally, although our findings were supported by *in vitro* experiments and *in vivo* models, the clinical validation cohort was relatively limited; therefore, larger independent patient cohorts are required to confirm the robustness and generalizability of IFI44L as a biomarker for CRT sensitivity.

In conclusion, our findings indicate that IFI44L expression is upregulated in responders receiving CRT. IFI44L overexpression enhanced CRT sensitivity in immunocompetent mice via upregulating CCL5 and promoting CD8^+^ T cell infiltration. These findings offer insights for the design of personalized therapeutic strategies and identifying potential strategies to sensitize CRC to CRT.

## Data Availability

The datasets presented in this study can be found in online repositories. The names of the repository/repositories and accession number(s) can be found in the article/**Supplementary Material**.
