# Supplementary material for: IFI44L modulates chemoradiotherapy sensitivity and CD8+ T cell infiltration in colorectal cancer
Source: Front Oncol. 2026 Aug 28;16:1786425. doi: 10.3389/fonc.2026.1786425 (PMC13561939; doi:10.3389/fonc.2026.1786425)
Supplement: Supplementary file 1 [file SupplementaryFile1.docx]

Supplementary Figure

**Supplementary Figure S1. In vivo treatment regimen.**

Cancer cells (HCT116, RKO or CT26) were subcutaneously injected into NSG or BALB/c mice and then treated with radiotherapy (4 Gy) and intraperitoneal chemotherapy (20 mg/kg 5-FU and 5 mg/kg of Oxaliplatin) or not at the indicated time points (n = 4-5 per group).

**Supplementary Figure S2. IFI44L overexpression does not affect colorectal cancer cell proliferation or chemoradiotherapy sensitivity in vitro.**

(A) CCK-8 proliferation assay of IFI44L-OE RKO and Vector RKO cells under untreated and irradiated conditions.

(B, C) Half-maximal inhibitory concentrations (IC_50_) of 5-fluorouracil (5-FU, panel B) and Oxaliplatin (OXA, panel C) in IFI44L-OE RKO and Vector RKO cell lines.

(D) CCK-8 proliferation assay of IFI44L-OE HCT116 and Vector HCT116 cells under untreated and irradiated conditions.

(E, F) IC_50_ values of 5-fluorouracil (5-FU, panel E) and Oxaliplatin (OXA, panel F) in IFI44L-OE HCT116 and Vector HCT116 cell lines.

**Supplementary Figure S3.** Receiver operating characteristic (ROC) curve analysis of IFI44L expression for predicting treatment response.

IFI44L expression demonstrated a favorable ability to discriminate responders (R) from non-responders (NR), with an area under the curve (AUC) of 0.774 (95% CI: 0.633–0.914).
